# High Sitting Time Is a Behavioral Risk Factor for Blunted Improvement in Depression Across 8 Weeks of the COVID-19 Pandemic in April–May 2020

**DOI:** 10.3389/fpsyt.2021.741433

**Published:** 2021-10-01

**Authors:** Jacob D. Meyer, John O'Connor, Cillian P. McDowell, Jeni E. Lansing, Cassandra S. Brower, Matthew P. Herring

**Affiliations:** ^1^Department of Kinesiology, Iowa State University, Ames, IA, United States; ^2^The Irish Longitudinal Study of Ageing (TILDA), Trinity College Dublin, The University of Dublin, Dublin, Ireland; ^3^Physical Activity for Health Research Cluster, Health Research Institute, Department of Physical Education and Sport Sciences, University of Limerick, Limerick, Ireland

**Keywords:** COVID-19, exercise, sedentary, screen time, depression, anxiety, positive mental health, longitudinal

## Abstract

The COVID-19 pandemic has elicited increased sedentary behaviors, decreased moderate-to-vigorous physical activity (MVPA), and worsened mental health, yet the longitudinal impact of these changes and their inter-relations remains unknown. Our purpose was to examine associations between changes in self-reported activity behaviors and mental health over an 8-week period following the COVID-19 outbreak. Participants from all 50 states and the District of Colombia were recruited through convenience and snowball sampling at baseline April 3–10, 2020. Prospective data from 2,327 US adults with ≥2 responses (63.8% female; 74.3% response rate) were collected weekly via online survey for eight consecutive weeks (April 3–June 3, 2020). Primary exposures were self-reported time spent sitting, viewing screens and in MVPA, with primary outcomes being depressive symptoms, anxiety symptoms, and positive mental health (PMH). A significant sitting-by-time interaction (*p* < 0.05) showed slightly higher marginal effects for depressive symptoms for the 90th-percentile of sitting time than the 10th-percentile at baseline (5.8 [95% confidence interval = 5.5–6.2] vs. 5.7 [5.4–6.1]), with the difference magnifying over time (week 8: 3.5 [3.2–3.9] vs. 2.7 [2.4–2.9]). No other interactions over time were significant. Screen time was negatively associated with PMH and positively associated with depressive and anxiety symptoms (*p* < 0.05). Sitting time was negatively associated with PMH (*p* < 0.05). Rapid changes in sitting patterns (e.g., due to a pandemic) may have lasting effects on depressive symptoms. Strategies targeting those most affected (i.e., young adults, females) and/or focused on reducing sitting time may be critical for preventing long-term mental health effects resulting from COVID-19 or other large-scale behavior changes in the general population.

## Introduction

The outbreak of the novel coronavirus, SARS-CoV-2, and associated disease (COVID-19) led to drastic changes in the US in March 2020. To reduce disease transmission, various mitigation strategies were employed with citizens instructed to self-isolate, quarantine, shelter in place and/or socially distance from others (1–3). Implementation of these COVID-19-related public health guidelines led to changes in how, when, and if, social, work, and health-related activities were performed.

Regular physical activity and low sedentary behaviors (sitting and screen time) are associated with better mental health (4–7); however, decreased physical activity and increased sedentary behaviors have been adverse by-products of adherence to COVID-19 related public health guidelines (8, 9). The majority of past research in this area has examined associations between movement behaviors and mental health over long time-periods with little-to-no data available for evaluating rapid alterations in behavior as occurred in response to the global pandemic. From our past research and that of others, cross-sectional or retrospective assessments of COVID-19-related decreased physical activity and increased sitting and screen time have been associated with higher anxiety symptoms, depressive symptoms, loneliness and stress, as well as lower positive mental health (PMH; a broad concept of mental well-being focused on positive constructs) (10–14). However, given the cross-sectional and/or retrospective recall designs of previous research, there is a need for prospective and longitudinal studies to determine the effects of pandemic-related changes in behavior on mental health over time.

Longitudinal data obtained during local COVID-19 outbreaks across the world show worsened mental health in those who contract the virus (15) and in the general population (16–19), and identifying modifiable factors that can play a preventive role is essential. A recent systematic review identified some demographic characteristics associated with higher cross-sectional psychological distress during the pandemic (being female, younger, living in a rural area, having a lower socioeconomic status) (20). Currently there is limited evidence regarding how pandemic-associated mental health changes are influenced by concurrent changes in physical activity and sedentary behaviors, particularly over time. Assessing these longitudinal associations is essential as many changes occurred rapidly, and recovery or improvement of participation in health-related behaviors over the ensuing weeks may critically influence the timing or extent of mental health improvements. The scant longitudinal evidence has significant limitations, including small samples, short follow-up, and/or data collected from narrow populations (21, 22).

Better understanding which changes in activity behaviors improve or mitigate deleterious changes in mental health over time may critically inform public health guidelines and policies should subsequent waves of COVID-19 or future pandemics occur. Associations between large-scale behavioral changes and mental health are not normally witnessed on such abbreviated time-scales, providing a unique opportunity to understand their interrelations if within-person data are also able to be collected on this rapid timeline. Thus, the present manuscript details a longitudinal examination of relationships between changes in self-reported physical activity, sitting time, and screen time with changes in depressive and anxiety symptoms and PMH in a robust sample of US adults from repeated measurements across 8 weeks, beginning shortly after the declaration of a national emergency (March 13th) regarding the COVID-19 outbreak in the US. This longitudinal data adds to previously identified associations between immediate changes in behavior and mental health (10) by prospectively assessing behavior across time in a large sample of US adults. As in previous research evaluating the longitudinal associations of physical activity and screen time with psychological functioning (23, 24), the present analysis employed Poisson generalized linear regression models to evaluate hypothesized associations between co-occurring changes in activity behaviors (primary independent variables) and in mental health (dependent variables) across time.

## Materials and Methods

### Procedure

This longitudinal study includes follow-up data from the *COVID-19 and Wellbeing Study* collected at Iowa State University, following approval as an exempt study by the University Institutional Review Board (IRB# 20-144-00). The aim of the present study was to evaluate the associations between changes in behavior and changes in mental health across time during the early phase of the initial wave of the pandemic in the United States in April and May, 2020. The full description of recruitment and the initial survey methods is provided elsewhere (10). Briefly, recruitment methods for the initial survey included: mass emails to Iowa State University students, faculty, staff, and alumni; snowball sampling; and posts to social media pages. Mass emails and posts included a link to an anonymous electronic survey for interested participants to read and consent to enrollment in the study and verify inclusion criteria of being ≥18 years of age and a current US resident.

The initial survey took 20–30 min and was completed by 3,133 adults from April 3rd−10th, 2020 who indicated interest in continued participation. Participants reported demographic information, health history, COVID-19-related public health guidelines being followed, health behaviors (i.e., amount of physical activity, sitting and screen time, smoking status, and alcohol consumption) prior and after making COVID-related behavioral changes, and mental health outcomes, including loneliness, social engagement, stress, distress, anxiety and depressive symptoms, and PMH. Participants had the opportunity to provide consent to be re-contacted to complete eight weekly abbreviated follow-up surveys, excluding demographic information and health history. Follow-up surveys were sent every 7 days from initial survey completion for 8 weeks.

### Study Sample

Participants in the analytic sample (*n* = 2,327; 74.3% participation rate of providing at least 2 complete sets of data across the 9 time points; 63.8% female) were fairly evenly dispersed across age categories from 18 to 74, with 234 participants aged ≥75 years, and were generally well-educated (85.0% college graduates or above), and overweight (body mass index = 26.9 ± 6.3 kg/m^2^; Table 1). All responses were used from each participant who provided data at least two times, resulting in 1,013–2,118 responses in the analysis at each of the 9 time points (baseline and weeks 1–8) with the mean number of responses provided per participant at 5.5 (median = 5). At baseline, mean ± SD activity times were: sitting time, 511 ± 203 min/day; screen time, 442 ± 218 min/day; and percent in MVPA categories, 0 min/week = 12%, 300 + min/week = 70%. Compared to the US adult population from 2019 Census Bureau data, the present sample had more females (63.8 vs. 50.8%), and a higher education level (college graduates 88.5 vs. 31.5%) (25), and, generally, were older, higher income, non-smoking, not quarantining, and had better mental health at baseline than those who did not complete any follow-ups (Supplementary Table 1).

**Table 1:** Participant demographics.

	**Analytical sample (*n* = 2,327)**
	**No. (%) or mean ± SD**
**Age (years)**	
18–24	304 (13.0%)
25–34	349 (15.0%)
35–44	312 (13.4%)
45–54	297 (12.8%)
55–64	392 (16.8%)
65–74	439 (18.8%)
75+	234 (10.0%)
**Sex**	
Male	842 (36.2%)
Female	1,485 (63.8%)
Body mass index (kg/m^2^)	26.9 ± 6.3
**Education**	
High school graduate	32 (1.4%)
Some college	236 (10.1%)
College graduate	966 (41.5%)
Graduate degree	1,093 (47.0%)
**Income**	
<$25,000	175 (7.5%)
$25,000–$34,999	82 (3.5%)
$35,000–$49,999	130 (5.6%)
$50,000–$74,999	342 (14.7%)
≥$75,000	1,439 (61.9%)
Not sure	59 (2.5%)
Decline	98 (4.2%)
**Smoker**	
Yes	48 (2.1%)
No	2,279 (97.9%)
**Drinker**	
Yes	1,623 (69.8%)
No	702 (30.2%)
Screen time (min/day)	441.9 ± 218.3
Sitting time (min/day)	511.2 ± 202.5
**MVPA (min/week)**	
0	258 (11.9%)
1–149	153 (7.0%)
150–299	245 (11.3%)
300+	1,521 (69.9%)
Beck depression inventory-II	9.1 ± 8.3
Beck anxiety inventory	7.0 ± 7.7
SWEMWBS-7	24.5 ± 4.6
**Quarantine**	
Yes	221 (9.5%)
No	2,106 (90.5%)
**Stay at home**	
Yes	1,198 (51.5%)
No	1,129 (48.5%)
**Social distancing**	
Yes	1,790 (76.9%)
No	537 (23.1%)

*kg/m^2^, kilograms/meter^2^; MVPA, moderate-to-vigorous physical activity; SD, standard deviation; SWEMWBS-7, Short Warwick-Edinburgh Mental Well-being Scale-7*.

### Measures

Baseline demographic information was self-reported, including age, gender, sex, race, education, marital and occupational status, height, weight, and current chronic health conditions. As public health guidelines and adherence to guidelines varied across cities and states, participants also reported current public health guidelines that they were following at each time-point, selecting zero or more from: quarantined, self-isolating, under shelter-in-place or stay-at-home order, and/or social distancing with Centers for Disease Control definitions provided for each guideline.

#### Exposures

After being provided with standard definitions, participants reported current average daily minutes of screen and sitting time, and minutes engaged in moderate and vigorous physical activity (reported separately) by responding to the following questions: “In the past week, how much time have you spent sitting daily?/What was your average daily screen-time per day this week?/How much time on an average day have you spent in [moderate/vigorous] activity this week?” Minutes of MVPA toward meeting physical activity guidelines were calculated as the sum of reported minutes of moderate activity and twice the reported number of minutes of vigorous activity consistent with the US Physical Activity Guidelines (26) and multiplied by 7 to get a weekly total. Participants were categorized based on MVPA level as inactive (0 min/week), low active (i.e., 1–149 min/week), active (i.e., 150–299 min/week), or high active (i.e., >300 min/week). As no threshold values for minutes of screen time or sitting time are available in the United States, sitting and screen time were modeled as continuous variables (minutes/day).

#### Outcomes

The 21-item Beck Anxiety Inventory (BAI) (27) assessed anxiety symptoms. Scores range from 0 to 63; higher scores indicate greater anxiety symptoms. Well-established psychometric properties include high internal consistency (α = 0.91 using all measurements in the present sample) and test-retest reliability (*r* = 0.75) (27) (*r* = 0.84 from baseline to week 1 in the present sample).

The 21-item Beck Depression Inventory-II (BDI-II) (28, 29), excluding the suicidality item, assessed depressive symptoms. Scores were divided by 20 and multiplied by 21 to calculate estimated total scores. Strong internal consistency (α = 0.93 in the present sample) and good test-retest reliability have previously been reported (*r* = 0.87 in the present sample).

The seven-item Short Warwick-Edinburgh Mental Well-being Scale (SWEMWBS-7) (14) was used to assess PMH. Total scores ranged from 7 to 35; higher scores indicate more positive mental health (30). Strong internal consistency (α = 0.91 in the present sample) and high test-retest reliability has previously been reported (*r* = 0.83) (30) (*r* = 0.82 in the present sample).

### Analysis

Analyses were performed using R (version 4.0.2) and R Studio (version 1.3). Participant characteristics were described by means and standard deviations for continuous variables and proportions for categorical variables. Case-wise deletion occurred if participants reported: >960 min/day of sitting or screen time, >10,080 min/week of MVPA (threshold equivalent to 8 h/day of vigorous and 8 h/day of moderate). Scores of <10 on SWEMWBS-7 were infrequent (0.56% of the responses) and were excluded in order to meet the normality of residuals assumption for the analytic models. Following removal of responses <10, visual inspection of a QQ plot confirmed improved agreement with the assumption of normality.

Data for BAI and BDI-II were non-normal (i.e., positively-skewed), so Poisson generalized linear mixed models were used to examine the trajectory of changes in activity-related behaviors with changes in depression (Model 1) and anxiety symptoms (Model 2). As PMH data were normally distributed, a standard linear mixed model was used (Model 3). In each model, physical activity, screen time, and sitting time (at each time point) and their interactions with time were used as primary factors of interest for each outcome. All models included fixed effects for age and sex, public health guidelines, time point (9 time points; weeks 0–8), and the interaction of time with each factor (excluding public heath restrictions as it did not improve any model). Random effects included an intercept and slope for each individual. Results are presented as estimates from the model: incidence rate ratio (IRR) for Models 1 and 2 and beta coefficient for Model 3. Mixed effects models were fitted using the lme4 package (31), marginal effects were produced using sjplot (32), and R-squared values were calculated using the MuMin package (33). Hypotheses were 2-sided, with level of significance for each model parameter set at 0.05.

## Results

### Associations Between Changes in Mental Health and Changes in Activity Behaviors

**Figure 1:**
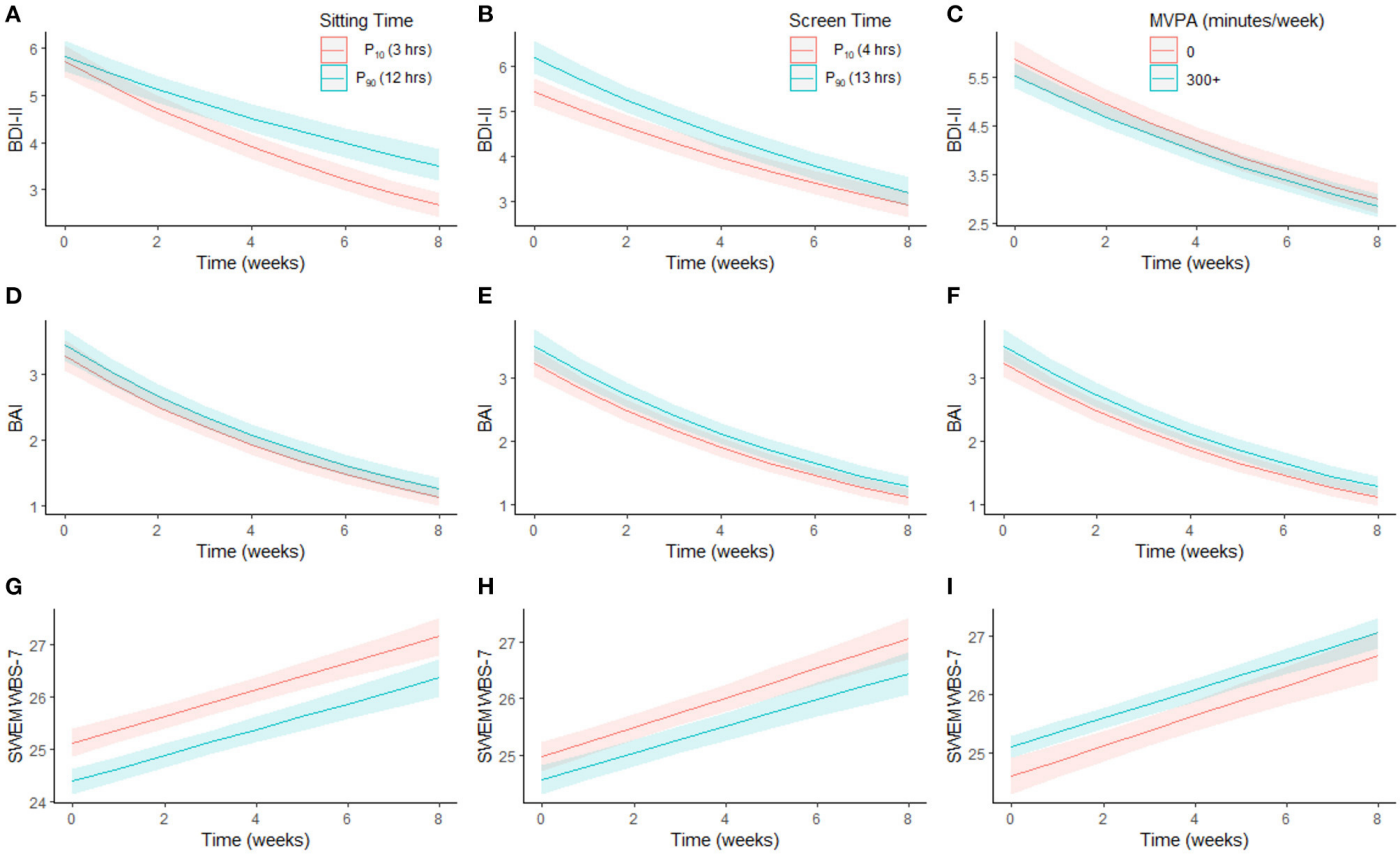
Association between changes in mental health over time by sitting time, screen time, and MVPA. Marginal effects plot showing trajectories in depressive symptoms **(A–C)**, anxiety symptoms **(D–F)** and positive mental health **(G–I)** over time by each exposure (sitting time, screen time, and MVPA), holding covariates at their mean (for continuous variables) or proportions (for factor variables). BAI, Beck Anxiety Inventory; BDI-II, Beck Depression Inventory II; MVPA, moderate-to-vigorous physical activity; SWEMWBS-7, Short Warwick-Edinburgh Mental Well-being Scale-7.

Full model results of linear mixed models for depressive and anxiety symptoms and PMH are presented in Table 2 (and Supplementary Tables 2–7). Predicted outcome scores at each time-point, while holding all covariates at their mean, for the 10th and 90th percentiles of sitting (i.e., 3 and 12 h) and screen time (i.e., 4 and 13 h), and for inactive (0 min/week) vs. high active (≥300 min/week) for physical activity, are presented in Figure 1. Marginal effects from the models, adjusted for age and sex, showed improvements in outcomes across all groups over time; however, there was some variation based on exposures.

**Table 2:** Full model results for depressive symptoms (BDI-II), anxiety symptoms (BAI), and positive mental health (SWEMWBS-7).

	**BDI-II IRR (95% CI)**	**BAI IRR (95% CI)**	**SWEMWBS-7 β (95% CI)**
**Fixed effects**			
Intercept	**2.97 (2.57, 3.43)**	**1.79 (1.50, 2.12)**	**27.21 (26.62, 27.80)**
Time	**0.92 (0.89, 0.94)**	**0.91 (0.88, 0.94)**	**0.25 (0.15, 0.34)**
Age (base = 75+ y)			
18–24 y	**3.50 (2.93, 4.17)**	**2.37 (1.92, 2.92)**	**−4.98 (−5.68**, **−4.28)**
25–34 y	**2.37 (2.00, 2.82)**	**1.71 (1.39, 2.10)**	**−3.26 (−3.93**, **−2.58)**
35–44 y	**2.16 (1.81, 2.57)**	**1.52 (1.24, 1.88)**	**−2.89 (−3.57**, **−2.21)**
45–54 y	**1.75 (1.47, 2.09)**	**1.29 (1.04, 1.59)**	**−2.10 (−2.79**, **−1.41)**
55–64 y	**1.22 (1.03, 1.44)**	0.97 (0.79, 1.18)	**−0.86 (−1.50**, **−0.21)**
65–74 y	1.06 (0.90, 1.25)	0.96 (0.79, 1.17)	−0.17 (−0.79, 0.45)
Sex (Female)	**1.38 (1.26, 1.51)**	**2.10 (1.88, 2.33)**	**−1.09 (−1.43**, **−0.74)**
MVPA (base = 0 min/week)			
1–149 (min/week)	1.01 (0.95, 1.08)	0.99 (0.91, 1.07)	0.05 (−0.34, 0.43)
150–299 (min/week)	0.97 (0.92, 1.03)	1.00 (0.93, 1.08)	0.14 (−0.20, 0.48)
300+ (min/week)	**0.94 (0.90, 0.99)**	0.95 (0.90, 1.01)	**0.50 (0.22, 0.79)**
Sitting time	1.01 (0.99, 1.03)	1.02 (0.99, 1.05)	**−0.30 (−0.41**, **−0.18)**
Screen time	**1.05 (1.03, 1.08)**	**1.03 (1.00, 1.06)**	**−0.15 (−0.27**, **−0.03)**
Quarantine	1.00 (0.97, 1.03)	0.99 (0.96, 1.03)	0.14 (−0.01, 0.28)
Shelter-in-place or stay at home order	1.00 (0.98, 1.02)	**1.03 (1.00, 1.05)**	**−0.12 (−0.22**, **−0.02)**
Social distancing	0.99 (0.97, 1.01)	**0.96 (0.94, 0.99)**	−0.06 (−0.17, 0.06)
Time: Age 18–24 y	0.98 (0.95, 1.01)	**0.93 (0.90, 0.97)**	**0.11 (0.01, 0.22)**
Time: Age 25–34 y	1.02 (0.99, 1.05)	**0.95 (0.91, 0.98)**	**−0.10 (−0.21**, **−0.00)**
Time: Age 35–44 y	1.01 (0.98, 1.04)	**0.94 (0.91, 0.98)**	−0.04 (−0.13, 0.06)
Time: Age 45–54 y	1.00 (0.97, 1.03)	0.97 (0.93, 1.00)	−0.02 (−0.11, 0.08)
Time: Age 55–64 y	**0.96 (0.94, 0.99)**	**0.94 (0.91, 0.98)**	0.00 (−0.09, 0.09)
Time: Age 65–74 y	0.97 (0.95, 1.00)	**0.95 (0.92, 0.98)**	0.00 (−0.08, 0.09)
Time: Female	**1.02 (1.01, 1.04)**	1.01 (0.99, 1.03)	0.03 (−0.02, 0.08)
Time: MVPA			
1–149 (min/week)	1.01 (0.99, 1.03)	**1.02 (1.00, 1.05)**	−0.02 (−0.11, 0.07)
150–299 (min/week)	1.00 (0.99, 1.02)	0.99 (0.97, 1.01)	0.01 (−0.07, 0.09)
300+ (min/week)	1.00 (0.99, 1.01)	1.01 (0.99, 1.02)	−0.02 (−0.08, 0.05)
Time: Sitting time	**1.01 (1.01, 1.02)**	1.00 (0.99, 1.01)	−0.00 (−0.03, 0.03)
Time: Screen time	1.00 (0.99, 1.00)	1.00 (1.00, 1.01)	−0.01 (−0.04, 0.02)
**Random effects**			
ID-intercept SD	0.92	1.08	3.59
ID-Time SD	0.12	0.14	0.37
ICC	0.46	0.54	0.78
Pseudo R^2^ (Fixed)	0.21	0.17	0.20
Pseudo R^2^ (Total)	0.96	0.94	0.85

*Bolded values indicate statistical significance of the individual term in the full model at p < 0.05*.

*BAI, Beck Anxiety Inventory, BDI-II, Beck Depression Inventory II; MVPA, moderate-to-vigorous physical activity; SWEMWBS-7, Short Warwick-Edinburgh Mental Well-being Scale-7; y, years*.

There was a significant sitting-by-time interaction for depressive symptoms. At baseline, there was no difference in predicted depressive symptoms between sitting for 3 and 12 h (3 h: 5.7 [5.4–6.1]; 12 h: 5.8 [5.5–6.2]); however, by week four, there was a significant difference depending on sitting time (3 h: 3.9 [3.7–4.2]; 12 h: 4.5 [4.2–4.8]; Figure 1A). This difference became more pronounced by week 8 (3 h: 2.7 [2.4–2.9]; 12 h: 3.5 [3.2–3.9]). Predicted anxiety symptoms (Figure 1D) did not differ by sitting time at baseline or across time. Across all time points, predicted PMH was lower/worse when sitting for 12 h compared to 3 h, but did not change differentially across time (Figure 1G).

At baseline, predicted depressive symptoms were higher in those viewing screens for 13 h compared to 4 h (4 h: 5.4 [5.1–5.8]; 13 h: 6.2 [5.9–6.6]); however, this difference was no longer apparent by week 3 (4 h: 4.3 [4.0–4.6]; 13 h: 4.8 [4.5–5.1]; Figure 1B), though the interaction between screen time and time was not significant. Predicted anxiety symptoms (Figure 1E) and PMH (Figure 1H) did not differ by screen time at baseline or across time. Similarly, predicted depressive (Figure 1C) and anxiety symptoms (Figure 1F) and PMH (Figure 1I) did not differ at baseline or across time by physical activity.

### Associations Between Changes in Mental Health and Age and Sex

**Figure 2:**
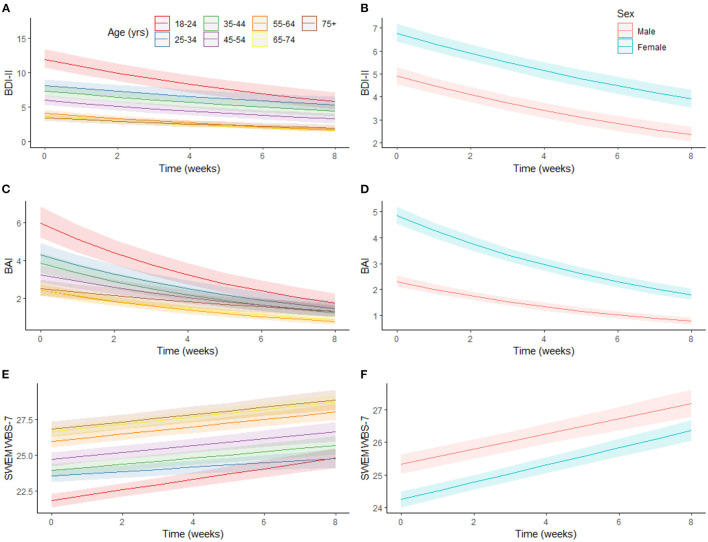
Association between changes in mental health over time by age, and sex. Marginal effects plot showing trajectories in depressive symptoms **(A,B)**, anxiety symptoms **(C,D)** and positive mental health **(E,F)** over time by age and gender, holding covariates at their mean (for continuous variables) or proportions (for factor variables). BAI, Beck Anxiety Inventory, BDI-II, Beck Depression Inventory II, SWEMWBS-7, Short Warwick-Edinburgh Mental Well-being Scale-7.

Predicted outcomes at each time point, while holding all covariates at their mean, for males and females and each age category are presented in Figure 2. Marginal effects from the models adjusting for sitting and screen time and physical activity showed improvements in outcomes across all groups over time. However, there was some variation based on sex and age.

Predicted depressive symptoms at baseline decreased with age, although changes across time did not vary by age (Figure 2A). Similar baseline results were observed for anxiety symptoms and age (Figure 2C). The relative decrease in anxiety was largest in the youngest age group (70.7%) and smallest in the oldest age group (48.0%). Predicted PMH at baseline increased with age (Figure 2E), although there was again no interaction with time.

Across all time points, females had higher predicted depressive (Figure 2B) and anxiety symptoms (Figure 2D) and lower predicted PMH (Figure 2F) than males; however, change in predicted depressive symptoms differed between males and females. Between baseline (males: 4.9 [4.6–5.3]; females: 6.8 [6.4–7.2]) and week 8 (males: 2.4 [2.1–2.7]; females: 3.9 [3.5–4.3]) the decrease relative to baseline scores was larger for males (males: 52.1%; females: 42.3%).

## Discussion

This study investigated how dynamic changes in physical activity, sitting time, and screen time in response to the COVID-19 pandemic were associated with changes in mental health across time. While the stringency of restrictions across the US was relatively consistent during this period (34), each mental health outcome improved over time showing a decay of the immediate response to the pandemic. However, key findings include that (1) sitting more was associated with a slower and limited improvement in depressive symptoms; (2) younger and/or female adults experienced worse mental health than other groups; and, (3) screen time and MVPA had limited impacts on mental health improvements. Implementation of COVID-19 public health restrictions resulted in abrupt changes in health-related behaviors that impacted mental health (10). The influence of changes in health-related behaviors over time, however, do not appear to be equal. Previous findings suggested that maintaining beneficial levels of health-related behaviors, including MVPA and screen time, may mitigate the immediate psychological impact of the pandemic. Though the present study showed that mental health generally improved in April and May after the acute implementation of mitigation strategies, high levels of sitting during this period were associated with blunted depressive symptom improvement which warrants further research.

Sedentary time substantially increased after implementation of COVID-19 public health restrictions (8). Moreover, associations between higher sedentary time and elevated depressive symptoms have been reported pre-(35) and post-COVID-19 (12). Yet, dynamic changes in sedentary behaviors across time are understudied, and longitudinal associations between changes in depressive symptoms and sedentary behaviors are largely unknown. Previous investigations have suggested that replacing sedentary time (total or prolonged) with higher intensity activity or sleep is associated with better mental health, including stress, depressive and anxiety symptoms (36, 37). Prospective cohort studies have also shown that sedentary activities, particularly mentally-passive sedentary activities, are associated with higher rates of major depression (38). Here, high sitting time during the period restrictions began to ease was associated with a blunted improvement in depressive symptoms, and the difference in depressive symptoms between low and high sitting at the beginning of June was larger than at the beginning of April. The magnitude of the difference between high (90th percentile) and low (10th percentile) sitting was small, yet indicative of potentially lasting effects of the pandemic that has seen large increases in depressive and anxiety symptoms (17, 18, 39). High levels of sitting may have limited the improvement in depressive symptoms that occurred across time, and continued high sitting time may be a key behavioral risk factor for lasting depressive symptoms. Data beyond 8 weeks is needed to confirm the present findings and determine the duration of relationships between high sitting and higher depressive symptoms. Future research should investigate if pandemic-associated increased sitting time attenuates over time, identify the longer-term mental and physical effects of pandemic-associated sitting time increases, examine whether the active or passive nature of sedentary time moderates these associations (38), and explore whether fractionating (i.e., breaking up) sitting time can improve depressive symptoms (40).

Consistent with previous evidence (17, 41–43), being female and/or younger were significantly and consistently associated with worse mental health outcomes across time, which did not completely return to comparator levels even after 8 weeks. Evidence from pandemic-related cross-sectional reports support associations between mental health and sex after COVID-19-related public health restrictions (22, 43–45) as well as poorer mental health in younger age groups (41–43). The lasting impact the pandemic may have on mental health in these populations is concerning. Though the present findings showed improved mental health over time in all groups (Figures 1, 2), understanding which factors contribute to initial impaired mental health among younger adults and/or females (e.g., changes to living situations or employment, limited social gatherings, larger access/time on media platforms) will be important for developing initiatives to curb acute and lasting mental health effects resulting from broad societal changes.

The present longitudinal findings suggest that screen time and MVPA may be less important than other health-behaviors for improved mental health during the easing of public health restrictions in the US. Yet, low screen time and high MVPA are associated with better mental health (7, 38), and these behaviors may have been helpful in blunting initial changes (10). Here, baseline mental health differences (depressive symptoms and PMH) were observed between those who engaged in large vs. small amounts of screen time. This is consistent with previous findings showing greater screen-time and social media exposure about COVID-19 were associated with higher anxiety and depressive symptoms (46, 47). Nonetheless, the magnitude of associations between screen-time and mental health diminished across time, which may reflect changes in the viewing of COVID-19-related information or a general beneficial effect of the passage of time on mental health. Strategies to limit pandemic-related media exposure could be useful for mental health overall, especially for young adults (or other vulnerable populations) who reported greater screen-time (47) and were most affected by the pandemic in the present study. While high MVPA (300+ min of MVPA/week) was associated with better mental health, there did not appear to be a large difference between high and no MVPA across time. The relatively low number of participants self-reporting being inactive (11.9% reported 0 baseline min, Table 1) may have limited statistical power to detect differences between MVPA groups. The overarching mental health response to the pandemic and easing of restrictions may have been greater than the beneficial effect of regular MVPA or low screen time.

## Limitations

There are potential limitations, including the convenience sample and follow-up rate. Participants in this study were not representative of the US population, in that they were predominantly white, with relatively high income and educational attainment, all of which are expected to be associated with better mental health. Therefore, the present results likely underestimated dynamic associations between activity behaviors and mental health that may be of an even greater magnitude in a more-vulnerable sample. The ~74% completion rate and robust sample completing ≥2 surveys (*n* = 2,327) reduces but does not eliminate the limitations associated with convenience sampling and attrition. Further, self-reported exposures and outcomes without secondary verification is a limitation, somewhat allayed by longitudinal analyses. Due to the variability in local policies (e.g., school closures, mask wearing), the present measure of currently-followed public health guidelines is unlikely to have captured the extent of the impact of the pandemic potentially introducing uncontrolled variability and leading to an underestimation of the present associations. Finally, the study design precludes causal inference, and reverse causation of pandemic-induced depressive symptoms resulting in higher sitting should also be considered.

## Conclusion

This longitudinal study of US adults showed generally improved mental health after initial implementation COVID-19 public health measures. However, high sitting time during this period was associated with a blunted recovery from elevated depressive symptoms and is of public health concern. With high rates of stress, anxiety, and depressive symptoms during this pandemic (15), modifiable factors associated with better mental health could be promising intervention targets. Strategies that target limiting overall sitting time may be important for preventing long-term mental health effects of lockdown periods or other periods of major workplace and societal shifts. Further, determining how long behavior changes in response to the pandemic last will be key to supporting long-term population physical and mental health.

## Data Availability Statement

The raw data supporting the conclusions of this article will be made available by the authors, without undue reservation.

## Ethics Statement

The studies involving human participants were reviewed and approved by Iowa State University Institutional Review Board. Written informed consent for participation was not required for this study in accordance with the national legislation and the institutional requirements.

## Author Contributions

JM, JL, and CB conceived of and participated in the design and coordination of the study, and prepared the data. JO'C, CM, and MH performed analyses. All authors were involved in the drafting of the manuscript, read and approved the final version and agree with the order of presentation of the authors.

## Funding

JO'C was employed by TILDA which was funded by the Health Research Board Ireland, Atlantic Philanthropies, the Irish Department of Health, Irish Life and Science Foundation Ireland. CM was funded by the Irish Research Council under the Government of Ireland Postdoctoral Programme. No funding bodies had any role in the design of the study, collection, analysis or interpretation of the data, or in the writing of the manuscript.

## Conflict of Interest

The authors declare that the research was conducted in the absence of any commercial or financial relationships that could be construed as a potential conflict of interest.

## Publisher's Note

All claims expressed in this article are solely those of the authors and do not necessarily represent those of their affiliated organizations, or those of the publisher, the editors and the reviewers. Any product that may be evaluated in this article, or claim that may be made by its manufacturer, is not guaranteed or endorsed by the publisher.
