# Supplementary material for: High Sitting Time Is a Behavioral Risk Factor for Blunted Improvement in Depression Across 8 Weeks of the COVID-19 Pandemic in April–May 2020
Source: Front Psychiatry. 2021 Oct 1;12:741433. doi: 10.3389/fpsyt.2021.741433 (PMC8519400; doi:10.3389/fpsyt.2021.741433)
Supplement: Supplementary file 1 [file Table_1.DOCX]

Supplementary Material

Supplementary data is presented for completeness and to provide opportunities to incorporate marginal estimates in future research.

**Supplemental Digital Table 1. Participant Demographics and Comparisons Between Analytic Sample and Excluded/Drop-Outs**

**Supplemental Digital Table 2. Adjusted Marginal Estimates for Depressive Symptoms (BDI-II)**

**Supplemental Digital Table 3. Adjusted Marginal Estimates for Anxiety Symptoms (BAI)**

**Supplemental Digital Table 4. Adjusted Marginal Estimates for Positive Mental Health (SWEMWBS-7)**

**Supplemental Digital Table 5. Unadjusted Marginal Estimates for Depressive Symptoms (BDI-II)**

**Supplemental Digital Table 6. Unadjusted Marginal Estimates for Anxiety Symptoms (BAI)**

**Supplemental Digital Table 7. Unadjusted Marginal Estimates for Positive Mental Health (SWEMWBS-7)**

**Supplemental Digital Table 1. Participant Demographics and Comparisons Between Analytic Sample and Excluded/Drop-Outs**

|  | | | |
| --- | --- | --- | --- |
|  | **Analytical sample (n=2327)** | **Excluded/drop-out (n=806)** | **Statistical test result** |
| **Age (y)** |  |  | χ^2^=97.1;  P<0.001 |
| 18-24 | 304 (13.0%) | 200 (24.8%) |  |
| 25-34 | 349 (15.0%) | 141 (17.5%) |  |
| 35-44 | 312 (13.4%) | 126 (15.6%) |  |
| 45-54 | 297 (12.8%) | 104 (12.9%) |  |
| 55-64 | 392 (16.8%) | 197 (12.0%) |  |
| 65-74 | 439 (18.8%) | 87 (10.7%) |  |
| 75+ | 234 (10.0%) | 51 (6.3%) |  |
| **Sex** |  |  | χ^2^=0.3;  P=0.602 |
| Male | 842 (36.2%) | 298 (37.3%) |  |
| Female | 1485 (63.8%) | 501 (62.7%) |  |
| **BMI (kg/m^2^)** | 26.9 ± 6.3 | 27.1 ± 6.5 | t=-0.59; P=0.557 |
| **Education** |  |  | χ^2^=81.3;  P<0.001 |
| HS graduate | 32 (1.4%) | 25 (3.1%) |  |
| Some college | 236 (10.1%) | 165 (20.7%) |  |
| College graduate | 966 (41.5%) | 331 (41.4%) |  |
| Graduate degree | 1093 (47.0%) | 278 (34.8%) |  |
| **Income** |  |  | χ^2^=29.6;  P<0.001 |
| <$25,000 | 175 (7.5%) | 98 (12.3%) |  |
| $25,000-$34,999 | 82 (3.5%) | 36 (4.5%) |  |
| $35,000-$49,999 | 130 (5.6%) | 46 (5.8%) |  |
| $50,000-$74,999 | 342 (14.7%) | 124 (15.5%) |  |
| ≥$75,000 | 1439 (61.9%) | 423 (52.9%) |  |
| Not sure | 59 (2.5%) | 30 (3.8%) |  |
| Decline | 98 (4.2%) | 42 (5.3%) |  |
| **Smoker** |  |  | χ^2^=8.2;  P=0.004 |
| **Yes** | 48 (2.1%) | 32 (4.0%) |  |
| **No** | 2279 (97.9%) | 767 (96.0%) |  |
| **Drinker** |  |  | χ^2^=0.1;  P=0.755 |
| **Yes** | 1623 (69.8%) | 551 (69.1%) |  |
| **No** | 702 (30.2%) | 246 (30.9%) |  |
| **Screen time (min/day)** | 441.9 ± 218.3 | 454.2 ± 230.0 | t=-1.3; P=0.192 |
| **Sitting time (min/day)** | 511.2 ± 202.5 | 505.4 ± 217.4 | t=0.7; P=0.512 |
| **MVPA (min/week)** |  |  | χ^2^=4.9;  P=0.178 |
| 0 | 258 (11.9%) | 105 (14.9%) |  |
| 1–149 | 153 (7.0%) | 51 (7.3%) |  |
| 150-299 | 245 (11.3%) | 76 (10.8%) |  |
| 300+ | 1521 (69.9%) | 469 (66.9%) |  |
| **BDI-II** | 9.1 ± 8.3 | 11.3 ± 9.5 | t=-6.1; P<0.001 |
| **BAI** | 7.0 ± 7.7 | 8.2 ± 8.9 | t=-3.3; P=0.001 |
| **SWEMWBS-7** | 24.5 ± 4.6 | 23.3 ± 5.1 | t=5.6; P<0.001 |
|  |  |  |  |
| **Quarantine** |  |  | χ^2^=11.0;  P=<0.001 |
| **Yes** | 221 (9.5%) | 110 (13.8%) |  |
| **No** | 2106 (90.5%) | 689 (86.2%) |  |
| **Stay at home** |  |  | χ^2^=0.6;  P=0.439 |
| **Yes** | 1198 (51.5%) | 398 (49.8%) |  |
| **No** | 1129 (48.5%) | 401 (50.2%) |  |
| **Social distancing** |  |  | χ^2^=0.7;  P=0.393 |
| **Yes** | 1790 (76.9%) | 627 (78.4%) |  |
| **No** | 537 (23.1%) | 172 (21.5%) |  |

**Supplemental Digital Table 2. Adjusted Marginal Estimates for Depressive Symptoms (BDI-II)**

|  |  | **Week 0/Baseline** | **Week 1** | **Week 2** | **Week 3** | **Week 4** | **Week 5** | **Week 6** | **Week 7** | **Week 8** |
| --- | --- | --- | --- | --- | --- | --- | --- | --- | --- | --- |
| Sitting - 10th percentile | Estimate | 5.72 | 5.20 | 4.73 | 4.30 | 3.91 | 3.55 | 3.23 | 2.93 | 2.67 |
|  | Lower CI | 5.40 | 4.92 | 4.47 | 4.05 | 3.66 | 3.31 | 2.98 | 2.69 | 2.42 |
|  | Upper CI | 6.05 | 5.49 | 5.00 | 4.56 | 4.16 | 3.81 | 3.49 | 3.20 | 2.94 |
|  | SE | 0.03 | 0.03 | 0.03 | 0.03 | 0.03 | 0.04 | 0.04 | 0.04 | 0.05 |
| Sitting - 90th percentile | Estimate | 5.83 | 5.47 | 5.14 | 4.82 | 4.52 | 4.25 | 3.98 | 3.74 | 3.51 |
|  | Lower CI | 5.52 | 5.18 | 4.86 | 4.54 | 4.24 | 3.95 | 3.68 | 3.42 | 3.18 |
|  | Upper CI | 6.16 | 5.78 | 5.43 | 5.11 | 4.83 | 4.56 | 4.32 | 4.09 | 3.87 |
|  | SE | 0.03 | 0.03 | 0.03 | 0.03 | 0.03 | 0.04 | 0.04 | 0.05 | 0.05 |
| Screen time - 10th percentile | Estimate | 5.43 | 5.02 | 4.65 | 4.30 | 3.97 | 3.68 | 3.40 | 3.14 | 2.91 |
|  | Lower CI | 5.13 | 4.75 | 4.39 | 4.05 | 3.73 | 3.42 | 3.14 | 2.88 | 2.64 |
|  | Upper CI | 5.75 | 5.31 | 4.91 | 4.56 | 4.24 | 3.95 | 3.68 | 3.43 | 3.21 |
|  | SE | 0.03 | 0.03 | 0.03 | 0.03 | 0.03 | 0.04 | 0.04 | 0.04 | 0.05 |
| Screen time - 90th percentile | Estimate | 6.20 | 5.71 | 5.25 | 4.83 | 4.45 | 4.09 | 3.76 | 3.46 | 3.19 |
|  | Lower CI | 5.85 | 5.40 | 4.96 | 4.55 | 4.17 | 3.81 | 3.47 | 3.17 | 2.88 |
|  | Upper CI | 6.57 | 6.04 | 5.56 | 5.13 | 4.75 | 4.40 | 4.08 | 3.79 | 3.52 |
|  | SE | 0.03 | 0.03 | 0.03 | 0.03 | 0.03 | 0.04 | 0.04 | 0.05 | 0.05 |
| 0 min MVPA | Estimate | 5.88 | 5.41 | 4.97 | 4.57 | 4.20 | 3.87 | 3.55 | 3.27 | 3.01 |
|  | Lower CI | 5.52 | 5.10 | 4.69 | 4.30 | 3.93 | 3.59 | 3.28 | 2.98 | 2.72 |
|  | Upper CI | 6.26 | 5.74 | 5.27 | 4.86 | 4.49 | 4.16 | 3.86 | 3.58 | 3.33 |
|  | SE | 0.03 | 0.03 | 0.03 | 0.03 | 0.03 | 0.04 | 0.04 | 0.05 | 0.05 |
| 300+ min MVPA | Estimate | 5.54 | 5.10 | 4.70 | 4.33 | 3.99 | 3.67 | 3.38 | 3.12 | 2.87 |
|  | Lower CI | 5.29 | 4.86 | 4.47 | 4.10 | 3.76 | 3.44 | 3.15 | 2.89 | 2.64 |
|  | Upper CI | 5.80 | 5.35 | 4.94 | 4.57 | 4.23 | 3.91 | 3.63 | 3.36 | 3.12 |
|  | SE | 0.02 | 0.02 | 0.03 | 0.03 | 0.03 | 0.03 | 0.04 | 0.04 | 0.04 |
| 18-24 y | Estimate | 11.91 | 10.87 | 9.92 | 9.06 | 8.27 | 7.55 | 6.90 | 6.30 | 5.75 |
|  | Lower CI | 10.64 | 9.67 | 8.75 | 7.90 | 7.13 | 6.42 | 5.77 | 5.18 | 4.65 |
|  | Upper CI | 13.32 | 12.23 | 11.26 | 10.39 | 9.61 | 8.89 | 8.24 | 7.65 | 7.10 |
|  | SE | 0.06 | 0.06 | 0.06 | 0.07 | 0.08 | 0.08 | 0.09 | 0.10 | 0.11 |
| 25-34 y | Estimate | 8.07 | 7.66 | 7.27 | 6.89 | 6.54 | 6.20 | 5.89 | 5.58 | 5.30 |
|  | Lower CI | 7.25 | 6.85 | 6.45 | 6.06 | 5.69 | 5.33 | 4.98 | 4.66 | 4.35 |
|  | Upper CI | 8.99 | 8.57 | 8.19 | 7.84 | 7.52 | 7.23 | 6.95 | 6.69 | 6.45 |
|  | SE | 0.05 | 0.06 | 0.06 | 0.07 | 0.07 | 0.08 | 0.09 | 0.09 | 0.10 |
| 35-44 y | Estimate | 7.33 | 6.87 | 6.44 | 6.03 | 5.66 | 5.30 | 4.97 | 4.65 | 4.36 |
|  | Lower CI | 6.56 | 6.12 | 5.69 | 5.28 | 4.89 | 4.52 | 4.18 | 3.86 | 3.56 |
|  | Upper CI | 8.20 | 7.72 | 7.29 | 6.90 | 6.54 | 6.21 | 5.90 | 5.61 | 5.34 |
|  | SE | 0.06 | 0.06 | 0.06 | 0.07 | 0.07 | 0.08 | 0.09 | 0.10 | 0.10 |
| 45-54 y | Estimate | 5.95 | 5.52 | 5.12 | 4.75 | 4.40 | 4.08 | 3.79 | 3.51 | 3.26 |
|  | Lower CI | 5.30 | 4.89 | 4.50 | 4.13 | 3.79 | 3.47 | 3.17 | 2.90 | 2.64 |
|  | Upper CI | 6.68 | 6.22 | 5.82 | 5.45 | 5.12 | 4.81 | 4.53 | 4.27 | 4.02 |
|  | SE | 0.06 | 0.06 | 0.07 | 0.07 | 0.08 | 0.08 | 0.09 | 0.10 | 0.11 |
| 55-64 y | Estimate | 4.14 | 3.72 | 3.33 | 2.99 | 2.68 | 2.40 | 2.15 | 1.93 | 1.73 |
|  | Lower CI | 3.74 | 3.34 | 2.97 | 2.64 | 2.35 | 2.08 | 1.84 | 1.62 | 1.43 |
|  | Upper CI | 4.60 | 4.14 | 3.73 | 3.38 | 3.06 | 2.78 | 2.53 | 2.30 | 2.09 |
|  | SE | 0.05 | 0.05 | 0.06 | 0.06 | 0.07 | 0.07 | 0.08 | 0.09 | 0.10 |
| 65-74 y | Estimate | 3.60 | 3.26 | 2.96 | 2.68 | 2.43 | 2.20 | 1.99 | 1.81 | 1.64 |
|  | Lower CI | 3.26 | 2.95 | 2.66 | 2.39 | 2.14 | 1.92 | 1.72 | 1.53 | 1.37 |
|  | Upper CI | 3.98 | 3.61 | 3.29 | 3.01 | 2.75 | 2.52 | 2.31 | 2.12 | 1.95 |
|  | SE | 0.05 | 0.05 | 0.05 | 0.06 | 0.06 | 0.07 | 0.08 | 0.08 | 0.09 |
| 75+ y | Estimate | 3.40 | 3.17 | 2.95 | 2.75 | 2.56 | 2.38 | 2.22 | 2.06 | 1.92 |
|  | Lower CI | 2.97 | 2.76 | 2.55 | 2.35 | 2.16 | 1.98 | 1.81 | 1.65 | 1.51 |
|  | Upper CI | 3.90 | 3.64 | 3.42 | 3.22 | 3.03 | 2.87 | 2.72 | 2.58 | 2.44 |
|  | SE | 0.07 | 0.07 | 0.08 | 0.08 | 0.09 | 0.10 | 0.10 | 0.11 | 0.12 |
| Male | Estimate | 4.91 | 4.48 | 4.09 | 3.73 | 3.40 | 3.10 | 2.83 | 2.58 | 2.35 |
|  | Lower CI | 4.56 | 4.15 | 3.77 | 3.42 | 3.09 | 2.80 | 2.53 | 2.28 | 2.06 |
|  | Upper CI | 5.29 | 4.83 | 4.43 | 4.06 | 3.73 | 3.44 | 3.17 | 2.92 | 2.69 |
|  | SE | 0.04 | 0.04 | 0.04 | 0.04 | 0.05 | 0.05 | 0.06 | 0.06 | 0.07 |
| Female | Estimate | 6.78 | 6.33 | 5.91 | 5.51 | 5.15 | 4.80 | 4.48 | 4.19 | 3.91 |
|  | Lower CI | 6.41 | 5.98 | 5.56 | 5.17 | 4.80 | 4.45 | 4.12 | 3.82 | 3.54 |
|  | Upper CI | 7.17 | 6.70 | 6.28 | 5.88 | 5.52 | 5.19 | 4.88 | 4.59 | 4.32 |
|  | SE | 0.03 | 0.03 | 0.03 | 0.03 | 0.04 | 0.04 | 0.04 | 0.05 | 0.05 |
| Note: Confidence intervals are 95% confidence intervals. Abbreviations: CI = confidence interval, SE = standard error, MVPA = moderate-to-vigorous physical activity. | | | | | | | | | | |

**Supplemental Digital Table 3. Adjusted Marginal Estimates for Anxiety Symptoms (BAI)**

|  |  | **Week 0/Baseline** | **Week 1** | **Week 2** | **Week 3** | **Week 4** | **Week 5** | **Week 6** | **Week 7** | **Week 8** |
| --- | --- | --- | --- | --- | --- | --- | --- | --- | --- | --- |
| Sitting - 10th percentile | Estimate | 3.27 | 2.87 | 2.51 | 2.20 | 1.93 | 1.69 | 1.48 | 1.30 | 1.13 |
|  | Lower CI | 3.05 | 2.68 | 2.35 | 2.05 | 1.78 | 1.55 | 1.34 | 1.16 | 1.00 |
|  | Upper CI | 3.51 | 3.07 | 2.69 | 2.36 | 2.08 | 1.84 | 1.63 | 1.44 | 1.28 |
|  | SE | 0.04 | 0.03 | 0.03 | 0.04 | 0.04 | 0.04 | 0.05 | 0.06 | 0.06 |
| Sitting - 90th percentile | Estimate | 3.44 | 3.03 | 2.67 | 2.36 | 2.08 | 1.83 | 1.61 | 1.42 | 1.25 |
|  | Lower CI | 3.21 | 2.84 | 2.50 | 2.20 | 1.92 | 1.68 | 1.46 | 1.27 | 1.11 |
|  | Upper CI | 3.68 | 3.24 | 2.86 | 2.53 | 2.25 | 2.00 | 1.78 | 1.59 | 1.42 |
|  | SE | 0.03 | 0.03 | 0.03 | 0.04 | 0.04 | 0.04 | 0.05 | 0.06 | 0.06 |
| Screen time - 10th percentile | Estimate | 3.23 | 2.83 | 2.48 | 2.17 | 1.90 | 1.66 | 1.45 | 1.27 | 1.12 |
|  | Lower CI | 3.01 | 2.65 | 2.32 | 2.02 | 1.76 | 1.52 | 1.32 | 1.14 | 0.99 |
|  | Upper CI | 3.46 | 3.02 | 2.65 | 2.32 | 2.05 | 1.81 | 1.60 | 1.42 | 1.26 |
|  | SE | 0.04 | 0.03 | 0.03 | 0.04 | 0.04 | 0.04 | 0.05 | 0.06 | 0.06 |
| Screen time - 90th percentile | Estimate | 3.50 | 3.09 | 2.72 | 2.40 | 2.12 | 1.87 | 1.65 | 1.45 | 1.28 |
|  | Lower CI | 3.26 | 2.89 | 2.55 | 2.24 | 1.96 | 1.71 | 1.49 | 1.30 | 1.13 |
|  | Upper CI | 3.76 | 3.31 | 2.92 | 2.58 | 2.29 | 2.04 | 1.82 | 1.63 | 1.45 |
|  | SE | 0.04 | 0.03 | 0.03 | 0.04 | 0.04 | 0.05 | 0.05 | 0.06 | 0.06 |
| 0 min MVPA | Estimate | 3.40 | 2.97 | 2.60 | 2.27 | 1.98 | 1.73 | 1.51 | 1.32 | 1.15 |
|  | Lower CI | 3.15 | 2.77 | 2.42 | 2.11 | 1.83 | 1.58 | 1.37 | 1.18 | 1.01 |
|  | Upper CI | 3.68 | 3.20 | 2.79 | 2.44 | 2.15 | 1.89 | 1.67 | 1.48 | 1.31 |
|  | SE | 0.04 | 0.04 | 0.04 | 0.04 | 0.04 | 0.05 | 0.05 | 0.06 | 0.07 |
| 300+ min MVPA | Estimate | 3.24 | 2.85 | 2.50 | 2.19 | 1.93 | 1.69 | 1.48 | 1.30 | 1.14 |
|  | Lower CI | 3.06 | 2.69 | 2.36 | 2.06 | 1.80 | 1.57 | 1.36 | 1.19 | 1.03 |
|  | Upper CI | 3.43 | 3.01 | 2.65 | 2.34 | 2.06 | 1.83 | 1.62 | 1.43 | 1.27 |
|  | SE | 0.03 | 0.03 | 0.03 | 0.03 | 0.04 | 0.04 | 0.04 | 0.05 | 0.05 |
| 18-24 y | Estimate | 5.97 | 5.12 | 4.39 | 3.77 | 3.23 | 2.77 | 2.38 | 2.04 | 1.75 |
|  | Lower CI | 5.21 | 4.45 | 3.79 | 3.21 | 2.71 | 2.29 | 1.93 | 1.62 | 1.36 |
|  | Upper CI | 6.83 | 5.89 | 5.09 | 4.42 | 3.85 | 3.36 | 2.93 | 2.57 | 2.25 |
|  | SE | 0.07 | 0.07 | 0.08 | 0.08 | 0.09 | 0.10 | 0.11 | 0.12 | 0.13 |
| 25-34 y | Estimate | 4.30 | 3.75 | 3.28 | 2.86 | 2.49 | 2.18 | 1.90 | 1.66 | 1.45 |
|  | Lower CI | 3.78 | 3.29 | 2.85 | 2.46 | 2.12 | 1.82 | 1.56 | 1.34 | 1.15 |
|  | Upper CI | 4.89 | 4.28 | 3.77 | 3.32 | 2.94 | 2.60 | 2.31 | 2.05 | 1.83 |
|  | SE | 0.07 | 0.07 | 0.07 | 0.08 | 0.08 | 0.09 | 0.10 | 0.11 | 0.12 |
| 35-44 y | Estimate | 3.84 | 3.33 | 2.88 | 2.50 | 2.17 | 1.88 | 1.63 | 1.41 | 1.22 |
|  | Lower CI | 3.35 | 2.90 | 2.49 | 2.14 | 1.83 | 1.56 | 1.33 | 1.13 | 0.96 |
|  | Upper CI | 4.39 | 3.82 | 3.34 | 2.92 | 2.57 | 2.26 | 2.00 | 1.76 | 1.56 |
|  | SE | 0.07 | 0.07 | 0.07 | 0.08 | 0.09 | 0.09 | 0.10 | 0.11 | 0.12 |
| 45-54 y | Estimate | 3.24 | 2.89 | 2.57 | 2.29 | 2.04 | 1.81 | 1.62 | 1.44 | 1.28 |
|  | Lower CI | 2.82 | 2.50 | 2.21 | 1.95 | 1.71 | 1.50 | 1.31 | 1.15 | 1.00 |
|  | Upper CI | 3.73 | 3.33 | 2.99 | 2.69 | 2.43 | 2.20 | 1.99 | 1.81 | 1.64 |
|  | SE | 0.07 | 0.07 | 0.08 | 0.08 | 0.09 | 0.10 | 0.11 | 0.12 | 0.13 |
| 55-64 y | Estimate | 2.44 | 2.11 | 1.82 | 1.58 | 1.36 | 1.18 | 1.02 | 0.88 | 0.76 |
|  | Lower CI | 2.15 | 1.86 | 1.60 | 1.37 | 1.17 | 0.99 | 0.85 | 0.72 | 0.61 |
|  | Upper CI | 2.76 | 2.39 | 2.08 | 1.82 | 1.59 | 1.39 | 1.23 | 1.08 | 0.95 |
|  | SE | 0.06 | 0.06 | 0.07 | 0.07 | 0.08 | 0.09 | 0.09 | 0.10 | 0.11 |
| 65-74 y | Estimate | 2.42 | 2.12 | 1.86 | 1.63 | 1.43 | 1.26 | 1.10 | 0.97 | 0.85 |
|  | Lower CI | 2.15 | 1.88 | 1.64 | 1.43 | 1.24 | 1.07 | 0.93 | 0.80 | 0.69 |
|  | Upper CI | 2.73 | 2.39 | 2.11 | 1.87 | 1.66 | 1.47 | 1.31 | 1.17 | 1.04 |
|  | SE | 0.06 | 0.06 | 0.06 | 0.07 | 0.07 | 0.08 | 0.09 | 0.10 | 0.11 |
| 75+ y | Estimate | 2.52 | 2.32 | 2.14 | 1.97 | 1.82 | 1.67 | 1.54 | 1.42 | 1.31 |
|  | Lower CI | 2.14 | 1.97 | 1.80 | 1.64 | 1.49 | 1.35 | 1.22 | 1.10 | 0.99 |
|  | Upper CI | 2.96 | 2.73 | 2.54 | 2.36 | 2.21 | 2.07 | 1.94 | 1.83 | 1.72 |
|  | SE | 0.08 | 0.08 | 0.09 | 0.09 | 0.10 | 0.11 | 0.12 | 0.13 | 0.14 |
| Male | Estimate | 2.31 | 2.02 | 1.76 | 1.54 | 1.34 | 1.17 | 1.03 | 0.90 | 0.78 |
|  | Lower CI | 2.11 | 1.84 | 1.60 | 1.39 | 1.20 | 1.04 | 0.89 | 0.77 | 0.66 |
|  | Upper CI | 2.53 | 2.21 | 1.94 | 1.71 | 1.50 | 1.33 | 1.18 | 1.04 | 0.92 |
|  | SE | 0.05 | 0.05 | 0.05 | 0.05 | 0.06 | 0.06 | 0.07 | 0.08 | 0.08 |
| Female | Estimate | 4.85 | 4.29 | 3.79 | 3.35 | 2.96 | 2.62 | 2.31 | 2.04 | 1.81 |
|  | Lower CI | 4.54 | 4.01 | 3.53 | 3.11 | 2.73 | 2.39 | 2.10 | 1.84 | 1.61 |
|  | Upper CI | 5.19 | 4.59 | 4.07 | 3.61 | 3.21 | 2.86 | 2.55 | 2.28 | 2.03 |
|  | SE | 0.03 | 0.03 | 0.04 | 0.04 | 0.04 | 0.05 | 0.05 | 0.05 | 0.06 |
| Note: Confidence intervals are 95% confidence intervals. Abbreviations: CI = confidence interval, SE = standard error, MVPA = moderate-to-vigorous physical activity. | | | | | | | | | | |

**Supplemental Digital Table 4. Adjusted Marginal Estimates for Positive Mental Health (SWEMWBS-7)**

|  |  | **Week 0/Baseline** | **Week 1** | **Week 2** | **Week 3** | **Week 4** | **Week 5** | **Week 6** | **Week 7** | **Week 8** |
| --- | --- | --- | --- | --- | --- | --- | --- | --- | --- | --- |
| Sitting - 10th percentile | Estimate | 25.13 | 25.38 | 25.64 | 25.89 | 26.14 | 26.39 | 26.65 | 26.90 | 27.15 |
|  | Lower CI | 24.87 | 25.14 | 25.41 | 25.66 | 25.90 | 26.14 | 26.36 | 26.58 | 26.80 |
|  | Upper CI | 25.39 | 25.62 | 25.86 | 26.12 | 26.38 | 26.65 | 26.93 | 27.22 | 27.51 |
|  | SE | 0.13 | 0.12 | 0.12 | 0.12 | 0.12 | 0.13 | 0.15 | 0.16 | 0.18 |
| Sitting - 90th percentile | Estimate | 24.39 | 24.64 | 24.88 | 25.13 | 25.38 | 25.62 | 25.87 | 26.12 | 26.37 |
|  | Lower CI | 24.15 | 24.41 | 24.66 | 24.90 | 25.13 | 25.36 | 25.58 | 25.79 | 26.00 |
|  | Upper CI | 24.64 | 24.87 | 25.11 | 25.36 | 25.62 | 25.89 | 26.17 | 26.45 | 26.73 |
|  | SE | 0.12 | 0.12 | 0.11 | 0.12 | 0.12 | 0.14 | 0.15 | 0.17 | 0.19 |
| Screen time - 10th percentile | Estimate | 24.97 | 25.23 | 25.49 | 25.75 | 26.02 | 26.28 | 26.54 | 26.80 | 27.07 |
|  | Lower CI | 24.71 | 24.99 | 25.26 | 25.52 | 25.77 | 26.02 | 26.25 | 26.48 | 26.70 |
|  | Upper CI | 25.22 | 25.46 | 25.72 | 25.98 | 26.26 | 26.54 | 26.83 | 27.13 | 27.43 |
|  | SE | 0.13 | 0.12 | 0.12 | 0.12 | 0.12 | 0.13 | 0.15 | 0.17 | 0.19 |
| Screen time - 90th percentile | Estimate | 24.57 | 24.80 | 25.04 | 25.27 | 25.51 | 25.74 | 25.98 | 26.21 | 26.45 |
|  | Lower CI | 24.30 | 24.56 | 24.81 | 25.04 | 25.26 | 25.47 | 25.68 | 25.88 | 26.07 |
|  | Upper CI | 24.83 | 25.04 | 25.27 | 25.51 | 25.76 | 26.01 | 26.28 | 26.55 | 26.83 |
|  | SE | 0.13 | 0.12 | 0.12 | 0.12 | 0.13 | 0.14 | 0.15 | 0.17 | 0.19 |
| 0 min MVPA | Estimate | 24.61 | 24.87 | 25.13 | 25.38 | 25.64 | 25.90 | 26.16 | 26.42 | 26.67 |
|  | Lower CI | 24.30 | 24.59 | 24.87 | 25.13 | 25.38 | 25.61 | 25.83 | 26.05 | 26.26 |
|  | Upper CI | 24.92 | 25.15 | 25.39 | 25.64 | 25.91 | 26.19 | 26.48 | 26.78 | 27.08 |
|  | SE | 0.16 | 0.14 | 0.13 | 0.13 | 0.14 | 0.15 | 0.17 | 0.19 | 0.21 |
| 300+ min MVPA | Estimate | 25.11 | 25.36 | 25.60 | 25.84 | 26.08 | 26.33 | 26.57 | 26.81 | 27.05 |
|  | Lower CI | 24.93 | 25.17 | 25.42 | 25.65 | 25.89 | 26.12 | 26.35 | 26.57 | 26.80 |
|  | Upper CI | 25.30 | 25.54 | 25.78 | 26.03 | 26.28 | 26.53 | 26.79 | 27.05 | 27.31 |
|  | SE | 0.10 | 0.09 | 0.09 | 0.10 | 0.10 | 0.11 | 0.11 | 0.12 | 0.13 |
| 18-24 y | Estimate | 21.84 | 22.21 | 22.58 | 22.95 | 23.32 | 23.69 | 24.06 | 24.43 | 24.80 |
|  | Lower CI | 21.37 | 21.75 | 22.12 | 22.47 | 22.81 | 23.14 | 23.47 | 23.78 | 24.10 |
|  | Upper CI | 22.31 | 22.67 | 23.04 | 23.43 | 23.83 | 24.23 | 24.65 | 25.07 | 25.50 |
|  | SE | 0.24 | 0.23 | 0.24 | 0.24 | 0.26 | 0.28 | 0.30 | 0.33 | 0.36 |
| 25-34 y | Estimate | 23.57 | 23.72 | 23.87 | 24.02 | 24.17 | 24.32 | 24.47 | 24.62 | 24.77 |
|  | Lower CI | 23.12 | 23.28 | 23.43 | 23.57 | 23.70 | 23.82 | 23.93 | 24.03 | 24.13 |
|  | Upper CI | 24.01 | 24.15 | 24.30 | 24.47 | 24.64 | 24.82 | 25.02 | 25.21 | 25.41 |
|  | SE | 0.23 | 0.22 | 0.22 | 0.23 | 0.24 | 0.26 | 0.28 | 0.30 | 0.33 |
| 35-44 y | Estimate | 23.93 | 24.15 | 24.37 | 24.59 | 24.81 | 25.03 | 25.25 | 25.47 | 25.69 |
|  | Lower CI | 23.48 | 23.70 | 23.92 | 24.13 | 24.33 | 24.51 | 24.70 | 24.87 | 25.05 |
|  | Upper CI | 24.38 | 24.59 | 24.81 | 25.05 | 25.29 | 25.54 | 25.80 | 26.06 | 26.33 |
|  | SE | 0.23 | 0.23 | 0.23 | 0.23 | 0.25 | 0.26 | 0.28 | 0.30 | 0.33 |
| 45-54 y | Estimate | 24.72 | 24.96 | 25.20 | 25.44 | 25.68 | 25.92 | 26.16 | 26.40 | 26.64 |
|  | Lower CI | 24.25 | 24.50 | 24.74 | 24.97 | 25.19 | 25.40 | 25.60 | 25.79 | 25.99 |
|  | Upper CI | 25.19 | 25.42 | 25.66 | 25.91 | 26.17 | 26.44 | 26.72 | 27.00 | 27.29 |
|  | SE | 0.24 | 0.23 | 0.23 | 0.24 | 0.25 | 0.27 | 0.29 | 0.31 | 0.33 |
| 55-64 y | Estimate | 25.96 | 26.22 | 26.48 | 26.74 | 26.99 | 27.25 | 27.51 | 27.77 | 28.02 |
|  | Lower CI | 25.56 | 25.82 | 26.08 | 26.33 | 26.57 | 26.80 | 27.02 | 27.24 | 27.46 |
|  | Upper CI | 26.37 | 26.62 | 26.88 | 27.15 | 27.42 | 27.71 | 28.00 | 28.29 | 28.59 |
|  | SE | 0.21 | 0.20 | 0.20 | 0.21 | 0.22 | 0.23 | 0.25 | 0.27 | 0.29 |
| 65-74 y | Estimate | 26.65 | 26.90 | 27.16 | 27.42 | 27.68 | 27.93 | 28.19 | 28.45 | 28.71 |
|  | Lower CI | 26.26 | 26.53 | 26.78 | 27.03 | 27.27 | 27.50 | 27.73 | 27.96 | 28.17 |
|  | Upper CI | 27.04 | 27.28 | 27.54 | 27.81 | 28.08 | 28.36 | 28.65 | 28.94 | 29.24 |
|  | SE | 0.20 | 0.19 | 0.19 | 0.20 | 0.21 | 0.22 | 0.23 | 0.25 | 0.27 |
| 75+ y | Estimate | 26.82 | 27.08 | 27.33 | 27.59 | 27.84 | 28.10 | 28.36 | 28.61 | 28.87 |
|  | Lower CI | 26.30 | 26.56 | 26.82 | 27.06 | 27.29 | 27.52 | 27.74 | 27.95 | 28.15 |
|  | Upper CI | 27.34 | 27.59 | 27.85 | 28.12 | 28.39 | 28.68 | 28.97 | 29.27 | 29.58 |
|  | SE | 0.27 | 0.26 | 0.26 | 0.27 | 0.28 | 0.30 | 0.32 | 0.34 | 0.36 |
| Male | Estimate | 25.33 | 25.56 | 25.80 | 26.03 | 26.27 | 26.50 | 26.73 | 26.97 | 27.20 |
|  | Lower CI | 25.03 | 25.27 | 25.51 | 25.74 | 25.96 | 26.17 | 26.38 | 26.58 | 26.78 |
|  | Upper CI | 25.63 | 25.85 | 26.09 | 26.33 | 26.58 | 26.83 | 27.09 | 27.35 | 27.62 |
|  | SE | 0.15 | 0.15 | 0.15 | 0.15 | 0.16 | 0.17 | 0.18 | 0.20 | 0.21 |
| Female | Estimate | 24.24 | 24.51 | 24.77 | 25.04 | 25.30 | 25.57 | 25.84 | 26.10 | 26.37 |
|  | Lower CI | 24.00 | 24.28 | 24.55 | 24.81 | 25.06 | 25.31 | 25.56 | 25.80 | 26.04 |
|  | Upper CI | 24.47 | 24.73 | 25.00 | 25.27 | 25.54 | 25.82 | 26.11 | 26.40 | 26.69 |
|  | SE | 0.12 | 0.12 | 0.11 | 0.12 | 0.12 | 0.13 | 0.14 | 0.15 | 0.17 |
| Note: Confidence intervals are 95% confidence intervals. Abbreviations: CI = confidence interval, SE = standard error, MVPA = moderate-to-vigorous physical activity. | | | | | | | | | | |

**Supplemental Digital Table 5. Unadjusted Marginal Estimates for Depressive Symptoms (BDI-II)**

|  |  | **Week 0/Baseline** | **Week 1** | **Week 2** | **Week 3** | **Week 4** | **Week 5** | **Week 6** | **Week 7** | **Week 8** |
| --- | --- | --- | --- | --- | --- | --- | --- | --- | --- | --- |
| Sitting - 10th percentile | Estimate | 5.34 | 4.89 | 4.48 | 4.10 | 3.76 | 3.44 | 3.15 | 2.88 | 2.64 |
|  | Lower CI | 5.06 | 4.64 | 4.24 | 3.87 | 3.53 | 3.22 | 2.93 | 2.66 | 2.42 |
|  | Upper CI | 5.63 | 5.16 | 4.73 | 4.34 | 4.00 | 3.68 | 3.39 | 3.13 | 2.88 |
|  | SE | 0.03 | 0.03 | 0.03 | 0.03 | 0.03 | 0.03 | 0.04 | 0.04 | 0.04 |
| Sitting - 90th percentile | Estimate | 6.14 | 5.74 | 5.36 | 5.01 | 4.68 | 4.38 | 4.09 | 3.82 | 3.57 |
|  | Lower CI | 5.82 | 5.44 | 5.08 | 4.73 | 4.40 | 4.08 | 3.79 | 3.52 | 3.26 |
|  | Upper CI | 6.48 | 6.05 | 5.67 | 5.31 | 4.99 | 4.69 | 4.41 | 4.16 | 3.91 |
|  | SE | 0.03 | 0.03 | 0.03 | 0.03 | 0.03 | 0.04 | 0.04 | 0.04 | 0.05 |
| Screen time - 10th percentile | Estimate | 5.20 | 4.79 | 4.42 | 4.08 | 3.76 | 3.47 | 3.20 | 2.95 | 2.72 |
|  | Lower CI | 4.93 | 4.55 | 4.19 | 3.85 | 3.53 | 3.24 | 2.97 | 2.72 | 2.49 |
|  | Upper CI | 5.48 | 5.05 | 4.67 | 4.32 | 4.00 | 3.71 | 3.44 | 3.20 | 2.97 |
|  | SE | 0.03 | 0.03 | 0.03 | 0.03 | 0.03 | 0.03 | 0.04 | 0.04 | 0.04 |
| Screen time - 90th percentile | Estimate | 6.42 | 5.93 | 5.48 | 5.07 | 4.68 | 4.33 | 4.00 | 3.70 | 3.42 |
|  | Lower CI | 6.08 | 5.62 | 5.19 | 4.78 | 4.39 | 4.04 | 3.70 | 3.40 | 3.12 |
|  | Upper CI | 6.77 | 6.26 | 5.79 | 5.37 | 4.99 | 4.64 | 4.32 | 4.02 | 3.75 |
|  | SE | 0.03 | 0.03 | 0.03 | 0.03 | 0.03 | 0.04 | 0.04 | 0.04 | 0.05 |
| 0 min MVPA | Estimate | 6.20 | 5.68 | 5.21 | 4.78 | 4.38 | 4.02 | 3.69 | 3.38 | 3.10 |
|  | Lower CI | 5.82 | 5.35 | 4.91 | 4.49 | 4.10 | 3.73 | 3.40 | 3.09 | 2.80 |
|  | Upper CI | 6.61 | 6.04 | 5.54 | 5.09 | 4.69 | 4.33 | 4.00 | 3.70 | 3.43 |
|  | SE | 0.03 | 0.03 | 0.03 | 0.03 | 0.03 | 0.04 | 0.04 | 0.05 | 0.05 |
| 300+ min MVPA | Estimate | 5.64 | 5.20 | 4.79 | 4.42 | 4.08 | 3.76 | 3.47 | 3.20 | 2.95 |
|  | Lower CI | 5.37 | 4.95 | 4.55 | 4.18 | 3.84 | 3.52 | 3.23 | 2.96 | 2.71 |
|  | Upper CI | 5.91 | 5.46 | 5.05 | 4.67 | 4.32 | 4.01 | 3.72 | 3.45 | 3.20 |
|  | SE | 0.02 | 0.03 | 0.03 | 0.03 | 0.03 | 0.03 | 0.04 | 0.04 | 0.04 |
| 18-24 y | Estimate | 13.16 | 12.04 | 11.02 | 10.08 | 9.22 | 8.44 | 7.72 | 7.06 | 6.46 |
|  | Lower CI | 11.80 | 10.73 | 9.73 | 8.81 | 7.96 | 7.18 | 6.47 | 5.83 | 5.25 |
|  | Upper CI | 14.69 | 13.52 | 12.48 | 11.54 | 10.69 | 9.92 | 9.21 | 8.56 | 7.95 |
|  | SE | 0.06 | 0.06 | 0.06 | 0.07 | 0.08 | 0.08 | 0.09 | 0.10 | 0.11 |
| 25-34 y | Estimate | 8.90 | 8.51 | 8.13 | 7.77 | 7.42 | 7.09 | 6.78 | 6.48 | 6.19 |
|  | Lower CI | 8.03 | 7.63 | 7.24 | 6.85 | 6.47 | 6.11 | 5.76 | 5.42 | 5.11 |
|  | Upper CI | 9.87 | 9.49 | 9.13 | 8.81 | 8.52 | 8.24 | 7.98 | 7.73 | 7.49 |
|  | SE | 0.05 | 0.06 | 0.06 | 0.06 | 0.07 | 0.08 | 0.08 | 0.09 | 0.10 |
| 35-44 y | Estimate | 7.76 | 7.30 | 6.87 | 6.46 | 6.08 | 5.72 | 5.38 | 5.06 | 4.76 |
|  | Lower CI | 6.95 | 6.50 | 6.07 | 5.65 | 5.26 | 4.88 | 4.53 | 4.20 | 3.89 |
|  | Upper CI | 8.66 | 8.20 | 7.77 | 7.39 | 7.03 | 6.70 | 6.39 | 6.10 | 5.82 |
|  | SE | 0.06 | 0.06 | 0.06 | 0.07 | 0.07 | 0.08 | 0.09 | 0.10 | 0.10 |
| 45-54 y | Estimate | 6.21 | 5.78 | 5.38 | 5.00 | 4.65 | 4.33 | 4.03 | 3.74 | 3.48 |
|  | Lower CI | 5.54 | 5.13 | 4.73 | 4.35 | 4.00 | 3.67 | 3.37 | 3.09 | 2.83 |
|  | Upper CI | 6.97 | 6.52 | 6.11 | 5.74 | 5.41 | 5.10 | 4.81 | 4.54 | 4.29 |
|  | SE | 0.06 | 0.06 | 0.07 | 0.07 | 0.08 | 0.08 | 0.09 | 0.10 | 0.11 |
| 55-64 y | Estimate | 4.18 | 3.74 | 3.35 | 2.99 | 2.68 | 2.40 | 2.15 | 1.92 | 1.72 |
|  | Lower CI | 3.77 | 3.36 | 2.99 | 2.65 | 2.35 | 2.07 | 1.83 | 1.62 | 1.43 |
|  | Upper CI | 4.63 | 4.16 | 3.75 | 3.38 | 3.06 | 2.77 | 2.51 | 2.28 | 2.07 |
|  | SE | 0.05 | 0.05 | 0.06 | 0.06 | 0.07 | 0.07 | 0.08 | 0.09 | 0.10 |
| 65-74 y | Estimate | 3.39 | 3.05 | 2.73 | 2.46 | 2.20 | 1.98 | 1.78 | 1.60 | 1.43 |
|  | Lower CI | 3.08 | 2.75 | 2.46 | 2.19 | 1.94 | 1.73 | 1.53 | 1.36 | 1.20 |
|  | Upper CI | 3.74 | 3.37 | 3.04 | 2.76 | 2.50 | 2.27 | 2.06 | 1.88 | 1.71 |
|  | SE | 0.05 | 0.05 | 0.05 | 0.06 | 0.06 | 0.07 | 0.08 | 0.08 | 0.09 |
| 75+ y | Estimate | 3.01 | 2.77 | 2.55 | 2.35 | 2.17 | 1.99 | 1.84 | 1.69 | 1.56 |
|  | Lower CI | 2.63 | 2.41 | 2.20 | 2.01 | 1.82 | 1.65 | 1.50 | 1.36 | 1.23 |
|  | Upper CI | 3.45 | 3.19 | 2.96 | 2.76 | 2.57 | 2.41 | 2.25 | 2.11 | 1.98 |
|  | SE | 0.07 | 0.07 | 0.08 | 0.08 | 0.09 | 0.10 | 0.10 | 0.11 | 0.12 |
| Male | Estimate | 4.00 | 3.62 | 3.28 | 2.97 | 2.69 | 2.44 | 2.21 | 2.00 | 1.81 |
|  | Lower CI | 3.70 | 3.35 | 3.02 | 2.72 | 2.44 | 2.19 | 1.97 | 1.77 | 1.58 |
|  | Upper CI | 4.32 | 3.92 | 3.57 | 3.25 | 2.97 | 2.71 | 2.48 | 2.27 | 2.08 |
|  | SE | 0.04 | 0.04 | 0.04 | 0.05 | 0.05 | 0.05 | 0.06 | 0.06 | 0.07 |
| Female | Estimate | 7.05 | 6.56 | 6.11 | 5.69 | 5.29 | 4.93 | 4.59 | 4.27 | 3.97 |
|  | Lower CI | 6.66 | 6.19 | 5.74 | 5.32 | 4.93 | 4.56 | 4.22 | 3.90 | 3.61 |
|  | Upper CI | 7.46 | 6.96 | 6.50 | 6.07 | 5.68 | 5.32 | 4.99 | 4.67 | 4.38 |
|  | SE | 0.03 | 0.03 | 0.03 | 0.03 | 0.04 | 0.04 | 0.04 | 0.05 | 0.05 |
| Note: Confidence intervals are 95% confidence intervals. Abbreviations: CI = confidence interval, SE = standard error, MVPA = moderate-to-vigorous physical activity. | | | | | | | | | | |

**Supplemental Digital Table 6. Unadjusted Marginal Estimates for Anxiety Symptoms (BAI)**

|  |  | **Week 0/Baseline** | **Week 1** | **Week 2** | **Week 3** | **Week 4** | **Week 5** | **Week 6** | **Week 7** | **Week 8** |
| --- | --- | --- | --- | --- | --- | --- | --- | --- | --- | --- |
| Sitting - 10th percentile | Estimate | 3.26 | 2.87 | 2.53 | 2.23 | 1.96 | 1.73 | 1.52 | 1.34 | 1.18 |
|  | Lower CI | 3.06 | 2.70 | 2.37 | 2.08 | 1.83 | 1.60 | 1.40 | 1.22 | 1.06 |
|  | Upper CI | 3.48 | 3.06 | 2.69 | 2.38 | 2.11 | 1.87 | 1.66 | 1.47 | 1.31 |
|  | SE | 0.03 | 0.03 | 0.03 | 0.03 | 0.04 | 0.04 | 0.04 | 0.05 | 0.05 |
| Sitting - 90th percentile | Estimate | 3.81 | 3.35 | 2.94 | 2.58 | 2.26 | 1.99 | 1.74 | 1.53 | 1.34 |
|  | Lower CI | 3.58 | 3.14 | 2.76 | 2.41 | 2.10 | 1.83 | 1.59 | 1.38 | 1.20 |
|  | Upper CI | 4.06 | 3.56 | 3.13 | 2.76 | 2.44 | 2.15 | 1.91 | 1.69 | 1.50 |
|  | SE | 0.03 | 0.03 | 0.03 | 0.03 | 0.04 | 0.04 | 0.05 | 0.05 | 0.06 |
| Screen time - 10th percentile | Estimate | 3.21 | 2.83 | 2.49 | 2.20 | 1.94 | 1.71 | 1.51 | 1.33 | 1.17 |
|  | Lower CI | 3.01 | 2.66 | 2.34 | 2.06 | 1.81 | 1.58 | 1.38 | 1.21 | 1.06 |
|  | Upper CI | 3.41 | 3.01 | 2.65 | 2.35 | 2.08 | 1.85 | 1.64 | 1.46 | 1.30 |
|  | SE | 0.03 | 0.03 | 0.03 | 0.03 | 0.04 | 0.04 | 0.04 | 0.05 | 0.05 |
| Screen time - 90th percentile | Estimate | 3.94 | 3.44 | 3.01 | 2.63 | 2.30 | 2.01 | 1.76 | 1.53 | 1.34 |
|  | Lower CI | 3.69 | 3.23 | 2.82 | 2.46 | 2.13 | 1.85 | 1.60 | 1.39 | 1.20 |
|  | Upper CI | 4.20 | 3.67 | 3.21 | 2.81 | 2.47 | 2.18 | 1.92 | 1.70 | 1.50 |
|  | SE | 0.03 | 0.03 | 0.03 | 0.03 | 0.04 | 0.04 | 0.05 | 0.05 | 0.06 |
| 0 min MVPA | Estimate | 3.84 | 3.33 | 2.88 | 2.50 | 2.17 | 1.88 | 1.63 | 1.41 | 1.22 |
|  | Lower CI | 3.55 | 3.10 | 2.69 | 2.32 | 2.00 | 1.72 | 1.48 | 1.26 | 1.08 |
|  | Upper CI | 4.15 | 3.58 | 3.09 | 2.69 | 2.34 | 2.05 | 1.80 | 1.58 | 1.39 |
|  | SE | 0.04 | 0.04 | 0.04 | 0.04 | 0.04 | 0.04 | 0.05 | 0.06 | 0.06 |
| 300+ min MVPA | Estimate | 3.46 | 3.04 | 2.67 | 2.35 | 2.07 | 1.82 | 1.60 | 1.41 | 1.24 |
|  | Lower CI | 3.27 | 2.87 | 2.52 | 2.21 | 1.93 | 1.69 | 1.48 | 1.29 | 1.12 |
|  | Upper CI | 3.66 | 3.22 | 2.83 | 2.50 | 2.21 | 1.96 | 1.73 | 1.54 | 1.36 |
|  | SE | 0.03 | 0.03 | 0.03 | 0.03 | 0.03 | 0.04 | 0.04 | 0.04 | 0.05 |
| 18-24 y | Estimate | 7.38 | 6.32 | 5.41 | 4.64 | 3.97 | 3.40 | 2.91 | 2.50 | 2.14 |
|  | Lower CI | 6.44 | 5.49 | 4.67 | 3.95 | 3.34 | 2.81 | 2.37 | 1.99 | 1.67 |
|  | Upper CI | 8.44 | 7.27 | 6.28 | 5.44 | 4.73 | 4.12 | 3.59 | 3.13 | 2.74 |
|  | SE | 0.07 | 0.07 | 0.08 | 0.08 | 0.09 | 0.10 | 0.11 | 0.12 | 0.13 |
| 25-34 y | Estimate | 5.32 | 4.66 | 4.07 | 3.57 | 3.12 | 2.73 | 2.39 | 2.09 | 1.83 |
|  | Lower CI | 4.69 | 4.08 | 3.55 | 3.07 | 2.66 | 2.29 | 1.97 | 1.70 | 1.46 |
|  | Upper CI | 6.04 | 5.31 | 4.68 | 4.14 | 3.67 | 3.26 | 2.90 | 2.58 | 2.30 |
|  | SE | 0.06 | 0.07 | 0.07 | 0.08 | 0.08 | 0.09 | 0.10 | 0.11 | 0.12 |
| 35-44 y | Estimate | 4.45 | 3.86 | 3.35 | 2.91 | 2.53 | 2.19 | 1.90 | 1.65 | 1.43 |
|  | Lower CI | 3.89 | 3.36 | 2.89 | 2.49 | 2.13 | 1.82 | 1.56 | 1.33 | 1.13 |
|  | Upper CI | 5.10 | 4.44 | 3.89 | 3.41 | 3.00 | 2.64 | 2.33 | 2.06 | 1.82 |
|  | SE | 0.07 | 0.07 | 0.08 | 0.08 | 0.09 | 0.09 | 0.10 | 0.11 | 0.12 |
| 45-54 y | Estimate | 3.70 | 3.30 | 2.94 | 2.61 | 2.33 | 2.07 | 1.85 | 1.64 | 1.46 |
|  | Lower CI | 3.22 | 2.85 | 2.52 | 2.22 | 1.95 | 1.71 | 1.50 | 1.31 | 1.15 |
|  | Upper CI | 4.26 | 3.81 | 3.42 | 3.08 | 2.78 | 2.51 | 2.27 | 2.06 | 1.87 |
|  | SE | 0.07 | 0.07 | 0.08 | 0.08 | 0.09 | 0.10 | 0.11 | 0.12 | 0.12 |
| 55-64 y | Estimate | 2.65 | 2.28 | 1.96 | 1.69 | 1.46 | 1.26 | 1.08 | 0.93 | 0.80 |
|  | Lower CI | 2.34 | 2.01 | 1.72 | 1.47 | 1.25 | 1.06 | 0.90 | 0.76 | 0.64 |
|  | Upper CI | 3.00 | 2.59 | 2.25 | 1.95 | 1.70 | 1.49 | 1.30 | 1.14 | 1.00 |
|  | SE | 0.06 | 0.07 | 0.07 | 0.07 | 0.08 | 0.09 | 0.09 | 0.10 | 0.11 |
| 65-74 y | Estimate | 2.28 | 1.98 | 1.73 | 1.50 | 1.30 | 1.13 | 0.99 | 0.86 | 0.75 |
|  | Lower CI | 2.02 | 1.76 | 1.52 | 1.31 | 1.13 | 0.97 | 0.83 | 0.71 | 0.61 |
|  | Upper CI | 2.57 | 2.24 | 1.96 | 1.72 | 1.51 | 1.33 | 1.17 | 1.04 | 0.92 |
|  | SE | 0.06 | 0.06 | 0.06 | 0.07 | 0.07 | 0.08 | 0.09 | 0.10 | 0.10 |
| 75+ y | Estimate | 2.07 | 1.89 | 1.73 | 1.58 | 1.45 | 1.33 | 1.21 | 1.11 | 1.02 |
|  | Lower CI | 1.76 | 1.60 | 1.46 | 1.32 | 1.19 | 1.07 | 0.96 | 0.86 | 0.77 |
|  | Upper CI | 2.43 | 2.23 | 2.06 | 1.91 | 1.77 | 1.64 | 1.53 | 1.43 | 1.34 |
|  | SE | 0.08 | 0.08 | 0.09 | 0.09 | 0.10 | 0.11 | 0.12 | 0.13 | 0.14 |
| Male | Estimate | 1.97 | 1.72 | 1.51 | 1.32 | 1.16 | 1.02 | 0.89 | 0.78 | 0.68 |
|  | Lower CI | 1.80 | 1.58 | 1.38 | 1.20 | 1.04 | 0.90 | 0.78 | 0.68 | 0.58 |
|  | Upper CI | 2.15 | 1.89 | 1.66 | 1.46 | 1.29 | 1.14 | 1.02 | 0.90 | 0.80 |
|  | SE | 0.05 | 0.05 | 0.05 | 0.05 | 0.06 | 0.06 | 0.07 | 0.07 | 0.08 |
| Female | Estimate | 4.92 | 4.32 | 3.79 | 3.33 | 2.92 | 2.56 | 2.25 | 1.98 | 1.73 |
|  | Lower CI | 4.62 | 4.05 | 3.54 | 3.09 | 2.70 | 2.35 | 2.05 | 1.79 | 1.55 |
|  | Upper CI | 5.24 | 4.60 | 4.05 | 3.58 | 3.16 | 2.79 | 2.47 | 2.19 | 1.94 |
|  | SE | 0.03 | 0.03 | 0.03 | 0.04 | 0.04 | 0.04 | 0.05 | 0.05 | 0.06 |
| Note: Confidence intervals are 95% confidence intervals. Abbreviations: CI = confidence interval, SE = standard error, MVPA = moderate-to-vigorous physical activity. | | | | | | | | | | |

**Supplemental Digital Table 7. Unadjusted Marginal Estimates for Positive Mental Health (SWEMWBS-7)**

|  |  | **Week 0/Baseline** | **Week 1** | **Week 2** | **Week 3** | **Week 4** | **Week 5** | **Week 6** | **Week 7** | **Week 8** |
| --- | --- | --- | --- | --- | --- | --- | --- | --- | --- | --- |
| Sitting - 10th percentile | Estimate | 25.47 | 25.72 | 25.97 | 26.22 | 26.47 | 26.71 | 26.96 | 27.21 | 27.46 |
|  | Lower CI | 25.25 | 25.51 | 25.76 | 26.01 | 26.25 | 26.49 | 26.73 | 26.96 | 27.18 |
|  | Upper CI | 25.69 | 25.93 | 26.17 | 26.42 | 26.68 | 26.94 | 27.20 | 27.47 | 27.74 |
|  | SE | 0.11 | 0.11 | 0.10 | 0.10 | 0.11 | 0.11 | 0.12 | 0.13 | 0.14 |
| Sitting - 90th percentile | Estimate | 24.18 | 24.43 | 24.68 | 24.93 | 25.18 | 25.43 | 25.67 | 25.92 | 26.17 |
|  | Lower CI | 23.96 | 24.22 | 24.47 | 24.72 | 24.95 | 25.19 | 25.42 | 25.64 | 25.87 |
|  | Upper CI | 24.40 | 24.64 | 24.88 | 25.14 | 25.40 | 25.66 | 25.93 | 26.21 | 26.48 |
|  | SE | 0.11 | 0.11 | 0.11 | 0.11 | 0.11 | 0.12 | 0.13 | 0.14 | 0.16 |
| Screen time - 10th percentile | Estimate | 25.39 | 25.65 | 25.91 | 26.17 | 26.44 | 26.70 | 26.96 | 27.22 | 27.48 |
|  | Lower CI | 25.17 | 25.44 | 25.71 | 25.97 | 26.22 | 26.47 | 26.72 | 26.96 | 27.20 |
|  | Upper CI | 25.61 | 25.86 | 26.12 | 26.38 | 26.65 | 26.92 | 27.20 | 27.48 | 27.77 |
|  | SE | 0.11 | 0.11 | 0.10 | 0.10 | 0.11 | 0.11 | 0.12 | 0.13 | 0.14 |
| Screen time - 90th percentile | Estimate | 24.17 | 24.43 | 24.68 | 24.94 | 25.19 | 25.45 | 25.70 | 25.95 | 26.21 |
|  | Lower CI | 23.94 | 24.21 | 24.47 | 24.72 | 24.96 | 25.20 | 25.44 | 25.67 | 25.90 |
|  | Upper CI | 24.40 | 24.64 | 24.89 | 25.15 | 25.42 | 25.69 | 25.96 | 26.24 | 26.52 |
|  | SE | 0.12 | 0.11 | 0.11 | 0.11 | 0.11 | 0.12 | 0.13 | 0.15 | 0.16 |
| 0 min MVPA | Estimate | 24.19 | 24.49 | 24.79 | 25.08 | 25.38 | 25.68 | 25.97 | 26.27 | 26.56 |
|  | Lower CI | 23.89 | 24.21 | 24.53 | 24.83 | 25.11 | 25.39 | 25.65 | 25.91 | 26.16 |
|  | Upper CI | 24.50 | 24.77 | 25.04 | 25.34 | 25.64 | 25.96 | 26.29 | 26.63 | 26.97 |
|  | SE | 0.16 | 0.14 | 0.13 | 0.13 | 0.13 | 0.15 | 0.16 | 0.18 | 0.21 |
| 300+ min MVPA | Estimate | 24.96 | 25.22 | 25.48 | 25.74 | 26.00 | 26.25 | 26.51 | 26.77 | 27.03 |
|  | Lower CI | 24.78 | 25.04 | 25.30 | 25.55 | 25.80 | 26.05 | 26.30 | 26.54 | 26.78 |
|  | Upper CI | 25.15 | 25.40 | 25.66 | 25.92 | 26.19 | 26.46 | 26.73 | 27.00 | 27.27 |
|  | SE | 0.09 | 0.09 | 0.09 | 0.09 | 0.10 | 0.10 | 0.11 | 0.12 | 0.13 |
| 18-24 y | Estimate | 21.41 | 21.81 | 22.21 | 22.62 | 23.02 | 23.42 | 23.82 | 24.23 | 24.63 |
|  | Lower CI | 20.96 | 21.37 | 21.76 | 22.15 | 22.52 | 22.89 | 23.25 | 23.60 | 23.95 |
|  | Upper CI | 21.86 | 22.26 | 22.67 | 23.09 | 23.52 | 23.96 | 24.40 | 24.85 | 25.31 |
|  | SE | 0.23 | 0.23 | 0.23 | 0.24 | 0.25 | 0.27 | 0.30 | 0.32 | 0.35 |
| 25-34 y | Estimate | 23.16 | 23.32 | 23.48 | 23.63 | 23.79 | 23.95 | 24.11 | 24.27 | 24.42 |
|  | Lower CI | 22.74 | 22.90 | 23.06 | 23.20 | 23.34 | 23.46 | 23.58 | 23.70 | 23.81 |
|  | Upper CI | 23.58 | 23.73 | 23.89 | 24.06 | 24.25 | 24.44 | 24.63 | 24.83 | 25.04 |
|  | SE | 0.21 | 0.21 | 0.21 | 0.22 | 0.23 | 0.25 | 0.27 | 0.29 | 0.31 |
| 35-44 y | Estimate | 23.72 | 23.94 | 24.16 | 24.38 | 24.61 | 24.83 | 25.05 | 25.28 | 25.50 |
|  | Lower CI | 23.27 | 23.50 | 23.72 | 23.93 | 24.13 | 24.33 | 24.51 | 24.70 | 24.88 |
|  | Upper CI | 24.16 | 24.38 | 24.60 | 24.84 | 25.08 | 25.33 | 25.59 | 25.86 | 26.12 |
|  | SE | 0.23 | 0.22 | 0.22 | 0.23 | 0.24 | 0.26 | 0.27 | 0.30 | 0.32 |
| 45-54 y | Estimate | 24.54 | 24.79 | 25.05 | 25.30 | 25.55 | 25.80 | 26.06 | 26.31 | 26.56 |
|  | Lower CI | 24.09 | 24.35 | 24.60 | 24.84 | 25.07 | 25.29 | 25.51 | 25.72 | 25.93 |
|  | Upper CI | 25.00 | 25.24 | 25.50 | 25.76 | 26.04 | 26.32 | 26.61 | 26.90 | 27.20 |
|  | SE | 0.23 | 0.23 | 0.23 | 0.24 | 0.25 | 0.26 | 0.28 | 0.30 | 0.32 |
| 55-64 y | Estimate | 25.99 | 26.26 | 26.54 | 26.82 | 27.10 | 27.38 | 27.65 | 27.93 | 28.21 |
|  | Lower CI | 25.59 | 25.88 | 26.15 | 26.42 | 26.68 | 26.93 | 27.18 | 27.43 | 27.67 |
|  | Upper CI | 26.38 | 26.65 | 26.93 | 27.22 | 27.52 | 27.82 | 28.12 | 28.44 | 28.75 |
|  | SE | 0.20 | 0.20 | 0.20 | 0.20 | 0.21 | 0.23 | 0.24 | 0.26 | 0.28 |
| 65-74 y | Estimate | 26.98 | 27.26 | 27.54 | 27.83 | 28.11 | 28.39 | 28.67 | 28.95 | 29.23 |
|  | Lower CI | 26.61 | 26.90 | 27.18 | 27.45 | 27.71 | 27.97 | 28.23 | 28.48 | 28.73 |
|  | Upper CI | 27.35 | 27.63 | 27.91 | 28.20 | 28.50 | 28.80 | 29.11 | 29.42 | 29.73 |
|  | SE | 0.19 | 0.19 | 0.19 | 0.19 | 0.20 | 0.21 | 0.22 | 0.24 | 0.26 |
| 75+ y | Estimate | 27.37 | 27.64 | 27.91 | 28.18 | 28.45 | 28.72 | 28.99 | 29.26 | 29.53 |
|  | Lower CI | 26.86 | 27.14 | 27.40 | 27.66 | 27.91 | 28.15 | 28.38 | 28.61 | 28.84 |
|  | Upper CI | 27.88 | 28.14 | 28.42 | 28.70 | 28.99 | 29.29 | 29.60 | 29.91 | 30.22 |
|  | SE | 0.26 | 0.26 | 0.26 | 0.26 | 0.28 | 0.29 | 0.31 | 0.33 | 0.35 |
| Male | Estimate | 26.11 | 26.36 | 26.61 | 26.86 | 27.11 | 27.37 | 27.62 | 27.87 | 28.12 |
|  | Lower CI | 25.81 | 26.07 | 26.32 | 26.56 | 26.80 | 27.04 | 27.27 | 27.50 | 27.73 |
|  | Upper CI | 26.40 | 26.65 | 26.90 | 27.16 | 27.43 | 27.69 | 27.97 | 28.24 | 28.52 |
|  | SE | 0.15 | 0.15 | 0.15 | 0.15 | 0.16 | 0.17 | 0.18 | 0.19 | 0.20 |
| Female | Estimate | 24.07 | 24.35 | 24.62 | 24.89 | 25.17 | 25.44 | 25.71 | 25.99 | 26.26 |
|  | Lower CI | 23.85 | 24.13 | 24.40 | 24.67 | 24.93 | 25.19 | 25.45 | 25.71 | 25.96 |
|  | Upper CI | 24.29 | 24.56 | 24.84 | 25.12 | 25.40 | 25.69 | 25.98 | 26.27 | 26.56 |
|  | SE | 0.11 | 0.11 | 0.11 | 0.11 | 0.12 | 0.13 | 0.13 | 0.14 | 0.15 |
| Note: Confidence intervals are 95% confidence intervals. Abbreviations: CI = confidence interval, SE = standard error, MVPA = moderate-to-vigorous physical activity. | | | | | | | | | | |
